# Manipulating rhizosphere microorganisms to improve crop yield in saline-alkali soil: a study on soybean growth and development

**DOI:** 10.3389/fmicb.2023.1233351

**Published:** 2023-09-20

**Authors:** Honglei Ren, Fengyi Zhang, Xiao Zhu, Sobhi F. Lamlom, Kezhen Zhao, Bixian Zhang, Jiajun Wang

**Affiliations:** ^1^Heilongjiang Academy of Agricultural Sciences, Soybean Research Institute, Harbin, China; ^2^Department of Plant Production, Faculty of Agriculture Saba Basha, Alexandria University, Alexandria, Egypt

**Keywords:** co-occurrence analysis, metagenomics, rhizosphere microorganisms, salinity, soybean phenotype

## Abstract

**Introduction:**

Rhizosphere microorganisms can effectively promote the stress resistance of plants, and some beneficial rhizosphere microorganisms can significantly promote the growth of crops under salt stress, which has the potential to develop special microbial fertilizers for increasing the yield of saline-alkali land and provides a low-cost and environmentally friendly new strategy for improving the crop yield of saline-alkali cultivated land by using agricultural microbial technology.

**Methods:**

In May 2022, a field study in a completely randomized block design was conducted at the Heilongjiang Academy of Agricultural Sciences to explore the correlation between plant rhizosphere microorganisms and soybean growth in saline-alkali soil. Two soybean cultivars (Hening 531, a salt-tolerant variety, and 20_1846, a salt-sensitive variety) were planted at two experimental sites [Daqing (normal condition) and Harbin (saline-alkali conditions)], aiming to investigate the performance of soybean in saline-alkali environments.

**Results:**

Soybeans grown in saline-alkali soil showed substantial reductions in key traits: plant height (25%), pod number (26.6%), seed yield (33%), and 100 seed weight (13%). This underscores the unsuitability of this soil type for soybean cultivation. Additionally, microbial analysis revealed 43 depleted and 56 enriched operational taxonomic units (OTUs) in the saline-alkali soil compared to normal soil. Furthermore, an analysis of ion-associated microbes identified 85 mOTUs with significant correlations with various ions. A co-occurrence network analysis revealed strong relationships between specific mOTUs and ions, such as Proteobacteria with multiple ions. In addition, the study investigated the differences in rhizosphere species between salt-tolerant and salt-sensitive soybean varieties under saline-alkali soil conditions. Redundancy analysis (RDA) indicated that mOTUs in saline-alkali soil were associated with pH and ions, while mOTUs in normal soil were correlated with Ca^2+^ and K^+^. Comparative analyses identified significant differences in mOTUs between salt-tolerant and salt-sensitive varieties under both saline-alkali and normal soil conditions. Planctomycetes, Proteobacteria, and Actinobacteria were dominant in the bacterial community of saline-alkali soil, with significant enrichment compared to normal soil. The study explored the functioning of the soybean rhizosphere key microbiome by comparing metagenomic data to four databases related to the carbon, nitrogen, phosphorus, and sulfur cycles. A total of 141 KOs (KEGG orthologues) were identified, with 66 KOs related to the carbon cycle, 16 KOs related to the nitrogen cycle, 48 KOs associated with the phosphorus cycle, and 11 KOs linked to the sulfur cycle. Significant correlations were found between specific mOTUs, functional genes, and phenotypic traits, including per mu yield (PMY), grain weight, and effective pod number per plant.

**Conclusion:**

Overall, this study provides comprehensive insights into the structure, function, and salt-related species of soil microorganisms in saline-alkali soil and their associations with salt tolerance and soybean phenotype. The identification of key microbial species and functional categories offers valuable information for understanding the mechanisms underlying plant-microbe interactions in challenging soil conditions.

## 1. Introduction

Soybean is an imperative economic crop, providing 30% of the world’s edible oil and 69% of protein (Zhang et al., 2016). Soybean [*Glycine max* (L.) Merrill] develops a vibrant microbial community near its roots, known as the rhizosphere. These microorganisms interact with the soybean plant, playing a crucial role in its growth and development (Hartmann et al., 2008). Furthermore, the performance of the soybean plants was influenced by various interacting biotic and abiotic factors. Cultivated soybean is usually salt-susceptible and requires genetic enhancement to improve the use of alkaline and salinized soils (Gao et al., 2022). Soil salinization is a worldwide threat because the increased level of salinization in recent years has resulted in food insecurity in numerous nations (Gao et al., 2022). Salinity stress affected soybean growth and agronomical characteristics such as nodulation, seed quality, and yield (Phang et al., 2008). Microorganisms in saline-alkali soil differ significantly from those in normal soil due to the higher salt content and pH levels. Additionally, the inhospitable conditions of saline-alkali soil pose challenges for microbial growth and survival, leading to alterations in microbial community composition and functioning. Furthermore, these differences influence soil fertility, nutrient cycling, and plant growth in saline-alkali environments (Burke et al., 2002; Han et al., 2020; Trivedi et al., 2020). The number of bacteria, fungi, and actinomycetes in saline-alkali soil is significantly lower than that in normal soil. In addition, the diversity of microbial species is also reduced due to the high salt content and alkalinity (Qu et al., 2020). The dominant species in saline-alkali soil are halophilic bacteria, which can tolerate high salt concentrations. These halophilic bacteria are usually Gram-negative rods or cocci and can produce exopolysaccharides to protect themselves from osmotic stress (Gao et al., 2022). In contrast, normal soils contain a variety of microorganisms such as bacteria, fungi, actinomycetes, protozoa, and algae. These organisms play important roles in nutrient cycling and decomposition of organic matter (Sharma et al., 2016; Sarkar et al., 2018; Wang et al., 2022). Plant roots possess a diverse microbial community primarily consisting of bacteria and fungi. These interactions between the plant and its microbial communities have a significant impact on the diversity and taxonomic structure of microbiomes. They also play a crucial role in facilitating essential processes within the host plant, such as nutrient acquisition and the ability to withstand changes in the biotic and abiotic environment (Compant et al., 2019; Pollak and Cordero, 2020).

Numerous studies have investigated the diversity and composition of rhizosphere microbiomes under different conditions, such as soybean genotype, growth stages, soil parameters, and geographical locations (Wang et al., 2022). These studies typically focus on identifying the abundance of different taxa, measuring alpha and beta diversities, and exploring correlations with environmental factors (Singh and Jha, 2016; Trivedi et al., 2020; Wang et al., 2022). However, traditional methods may not fully capture the complex interactions that microorganisms develop within their natural habitat, including mutualism, competition, parasitism, and commensalism with the host (Yeoh et al., 2017). To address this limitation, microbial network analysis, particularly co-occurrence networks, has gained popularity in studying microbial community structures in various environments, such as soil and the ocean. Co-occurrence networks reveal non-random patterns of microbial associations and often exhibit modular organization, representing groups of taxa with overlapping ecological niches (Zhang et al., 2018). These networks allow us to identify densely interconnected nodes, which can be interpreted as taxa that are more closely related and are likely to interact within specific niches (Zhang et al., 2016). While interpreting these networks can be challenging, they provide insights into hub species and their interactions within a particular niche (Chang et al., 2019). Similar network-based approaches have been used to determine protein interactions and predict gene functions from RNA-seq experiments by identifying gene significance and hub genes (Zhong et al., 2019). In microbial studies, co-occurrence networks using *in silico* sequence data have also been employed to identify taxa significance and keystone species (Chen et al., 2020). The advent of high-throughput sequencing technologies has facilitated rapid and efficient analysis of microbial communities by directly sequencing the 16S ribosomal RNA (rRNA) gene, which is a small subunit rRNA gene in prokaryotes (Sola-Leyva et al., 2021). Abiotic stress is thought to be the global source of 50% of yearly yield losses in key crops (Chatterton and Punja, 2010; Lamlom et al., 2020). Therefore, it is crucial to find additional microorganisms that can withstand diverse types of abiotic stress in the field, such as salt, alkaline, drought, etc. These bacteria might then be used as bioagents. These days, various salt-tolerant plant growth-promoting rhizobacteria (PGPR), including Bacillus, Rhizobium, Pseudomonas, and others, have demonstrated remarkable potential for reducing the salinity stress in many crops (Han et al., 2021). It has been observed that Bacillus, Pseudomonas, and Rhizobacteria mitigate the negative effects of salt stress in soybean (Khan et al., 2019; Abulfaraj and Jalal, 2021; Abdelghany et al., 2022). Actinobacteria have been evaluated for their ability to prime the alkaline or saline-alkali tolerance of soybean in soda-alkaline soils. Alkaline or soda saline-alkali soils are predominantly formed of NaHCO_3_/Na_2_CO_3_ with excess Na+, HCO_3_^–^/CO_3_^2–^, a high pH (>8.5), and weak soil structure, which causes greater plant development harm than saline soils (Gao et al., 2022). Halotolerant soil bacteria are a potential approach for dealing with salt stressors in edible crops. It may be able to alleviate the destructive consequences of salinity by generating a variety of growth-regulating PGP compounds (Sharma et al., 2016; Singh and Jha, 2016; Aslam and Ali, 2018; Sarkar et al., 2018; El-Sorady et al., 2022). The genetic assortment of ST-PGPR isolated from the wheat rhizosphere revealed that most of the isolates belonged to the species Bacillus and could survive up to 8% NaCl (Upadhyay et al., 2012). A total of 305 bacterial strains, and 162 of them were evaluated for salt tolerance up to a concentration of 150 g/l NaCl. Most of the isolates bacterial strains also showed the ability to improve salt tolerance, growth, and yield of rice under salt-stress conditions (Zhang et al., 2016). This research aimed to explore the potential of rhizosphere microorganisms in enhancing plant stress resistance, particularly under salt stress conditions. The study sought to identify beneficial rhizosphere microorganisms that could promote crop growth in saline-alkali soil, leading to the development of specialized microbial fertilizers for increasing yields on such land. By leveraging agricultural microbial technology, the study aimed to introduce a cost-effective and eco-friendly strategy to enhance crop yields in saline-alkali cultivated areas.

## 2. Materials and methods

### 2.1. Plant materials and soil sampling

In the summer season of 2022, a field study was conducted in the Daqing Branch of the Heilongjiang Academy of Agricultural Sciences to study the resistance of soybean to salt and alkali conditions. Two soybean cultivars, namely salt-tolerant (Heinong531) (ST) and salt-sensitive (20_1846) (SS), were planted at two experimental sites, the first site is Daqing located at (125°19′16.59″E, 46°62′5.31″N) and 147.5 m asl, whereas the second site is Harbin located at (126°51′41.91″E, 45°50′37.82″N) and 174 m asl. The initial physicochemical properties of the soil were determined during both seasons (Table 1), a kilogram of soil samples was collected at a depth of 20 cm at the two experimental sites during the flowering period (Kremer, 2019). The environmental conditions during the field experiments were shown in Table 2. All agricultural practice was done as recommended in the experimental sites.

**TABLE 1:** The soil chemical properties at a depth of 20 cm.

Location	Available nitrogen (mg/kg)	Available phosphorus (mg/kg)	Available potassium (mg/kg)	Total nitrogen (%)	Total phosphorus (%)	Total potassium (%)	Organic matter (g/kg)
Daqing	135.27	44.26	116.70	0.18	0.08	2.48	28.05
Harbin	234.14	437.88	381.07	0.17	0.08	2.71	32.20

**TABLE 2:** Meteorological data during the experiment season.

Months	April		May		June		July		August		September		October	
**City**	**Harbin**	**Daqing**	**Harbin**	**Daqing**	**Harbin**	**Daqing**	**Harbin**	**Daqing**	**Harbin**	**Daqing**	**Harbin**	**Daqing**	**Harbin**	**Daqing**
Average temperature (°C)	11.3	24.3	10.5	32.2	18	30.9	23.1	33.3	21.7	28.5	18.7	27.8	9.9	21.5
Precipitation	7.5	6.5	42	68.6	53.6	71.5	48.4	35	26.4	106.4	20.1	63.4	1.5	12.4
Sunshine hours	63.6	100.8	52.8	107.6	65	100.7	47.9	60.9	35.3	53.6	69.3	59.2	61.1	52.4

### 2.2. Growth characters

During the field experiments, we investigated multiple growth parameters to evaluate the influence of saline-alkali soil on soybean growth and productivity. We examined growth and yield factors in twenty soybean plants, encompassing measurements such as stem length, bottom pod height, effective pod number per plant, number of grains per plant, grain weight per plant, 100 seed weight, and per mu yield.

### 2.3. Soil ion concentration detection

Atomic absorption spectrometry (PerkinElmer Analyst 700; PerkinElmer, Norwalk, CT, USA) was used to measure soil Na^+^ concentration and plants Na^+^ and K^+^ concentrations (Crompton, 2006). The concentrations of the Cl anions in the soil were measured by ion chromatography apparatus (ICS-3000; Dione, Sunnyvale, CA, USA). As well as, the molybdenum-antimony colorimetric method was used to analyze the soil available phosphorus (AP), and a pH meter was used to measure the soil pH (Crompton, 2006).

### 2.4. DNA extraction and quality inspection

Twelve samples of soybean rhizosphere soil, including 6 samples of salt-tolerant rhizosphere soil and 6 samples of sensitive rhizosphere soil from the saline-alkali soil and normal soil. Total genomic DNA was extracted from 300 mg of each rhizosphere soil sample, using the Fast DNA Spin Kit for Soil (MP Biomedicals, Santa Ana, CA, USA). Extracted DNA was checked in 1% agarose gels run in 0.5 × Tris–acetate–EDTA (TAE) buffer (100 V, 15 min) to assess the quality of the extractions. DNA concentration was measured using a Qubit^®^ dsDNA Assay Kit in a Qubit^®^ 2.0 Fluorometer (Life Technologies, California, USA). DNA amounts >1 μg were used to construct the library (Fatima et al., 2014). Each analysis was performed with three biological replicates. Additionally, control samples were included in the sequencing and analysis to ensure robust comparisons and accurate interpretation of the results.

### 2.5. Shotgun metagenomic sequencing

By generating 10 Gb of raw data per sample we aimed to achieve a robust representation of the microbial genetic material, allowing for a thorough analysis of the microbial composition and functional attributes. This sequencing depth enabled the detection of a wide range of microbial taxa and genes, facilitating an in-depth exploration of the relationships between microbial communities, soil conditions, and soybean growth characteristics. For this study, a metagenomic approach was employed, utilizing a sequencing depth of 10 Gb per sample. To facilitate statistical analysis, we defined each species level as an mOTU based on species classification. Shotgun metagenomic libraries were constructed using a TruSeq DNA Sample Preparation kit (Illumina, San Diego, CA, USA) and sequenced on an Illumina NovaSeq sequencer (Illumina) to generate 10 Gb of raw data per sample. For the preparation of libraries, an initial amount of 25–50 ng of DNA was extracted from the samples collected at each site. These DNA samples underwent a process of fragmentation and the addition of adapter sequences. These adapters were then used in a limited-cycle PCR, and unique indices were incorporated into each sample. After the library preparation, the final concentration of the libraries was determined. The average size of the libraries was assessed using the Agilent 2100 Bioanalyzer, manufactured by Agilent Technologies in the USA. The DNA libraries obtained were subsequently combined in an equal molar ratio of 0.7 nM. Finally, the pooled libraries underwent 300 cycles of pair-end sequencing using the Illumina NovaSeq system. Quality control was performed to ensure that libraries had a concentration greater than 3 ng/μl. The obtained input FASTQ files were filtered and 3′ ends-trimmed by quality, using FASTQ (version 0.12.4) (Chen et al., 2018). Human host reads were removed by mapping the reads against the reference human genome H38, by using knead data (version 0.10.0) (McIver et al., 2018). Functional annotation was carried out with HMMER against the Kyoto Encyclopedia of Genes and Genomes (KEGG) database, version 2016 (Kanehisa and Goto, 2000) to obtain the functional subcategory, route, and annotation of the genes. Taxonomic annotation was implemented with kraken2 on the metagenomics reads (Sola-Leyva et al., 2021).

### 2.6. Bioinformatics and statistical analysis

Statistical analyses of phenotype were performed using GraphPad Prism (version 7.0) with a two-tailed test. Alpha diversity was assessed using the ACE, Chao1, and Richness indexes. Beta diversity was calculated with the vegan package (version 2.5-7) in R (version R-4.0.5), using the Bray–Curtis’s method as the distance measure and principal coordinate analysis (PCoA). ANOSIM tests were performed to identify differences in β-diversity between the two groups. The key species and function of the gene responsible for the distinction between the two groups were identified using edge R (*P*-value < 0.05, FDR < 0.2). Only *P*-value < 0.05 and FDR < 0.2 were considered significantly enriched. Redundancy Analysis (RDA) and Mantel tests were performed in R (version R-4.0.5) using the vegan package (version 2.5-7). These analyses aimed to explore relationships and associations between variables. Additionally, the correlation and co-occurrence network analysis were based on the Pearson method with Benjamini-Hochberg (BH) adjustment. A heatmap was generated to visualize the correlation results, with yellow boxes indicating positive correlations and green boxes representing negative correlations based on Pearson’s correlation coefficient. Statistical significance levels were indicated by asterisks (*, **, and ***) to denote *P*-values of less than 0.05, 0.01, and 0.001, respectively. The thickness of the lines in the co-occurrence network represented the strength of the correlation, and the line color indicated a positive or negative correlation. Dot size in the co-occurrence network represented the relative abundance of the species. To identify key bacterial taxa and functional genes responsible for discriminating between the two groups Welch’s *t*-test method in STAMP was employed. Significance was determined based on *p*-values corrected for multiple testing, with a threshold of less than 0.05.

## 3. Results

### 3.1. Effect of saline-alkali soil on soybean growth

**FIGURE 1:**
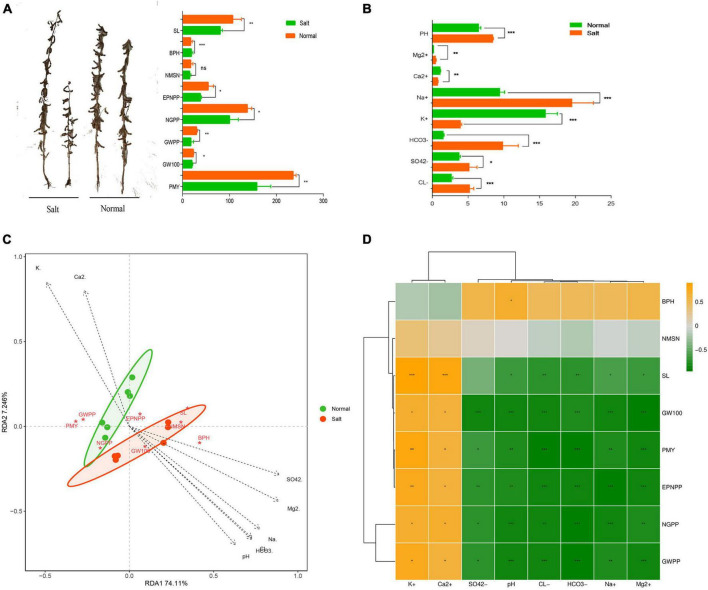
Effects of salinity soil on soybean growth. **(A)** Soybean phenotype data statistics between salinity soil and normal soil by *t*-test method of two-tailed. **(B)** The difference of ion concentration and PH between salinity and normal soil. Data statistics by *t*-test method of two-tailed. **(C)** Redundancy analysis (RDA) based on the phenotype of samples under the salinity condition (Mantel test, *n* = 12, *P* < 0.05). **(D)** Correlation analysis between differential phenotype and ion concentration. Yellow boxes represent positive correlations, while green boxes represent negative correlations (Pearson’s correlation). All black asterisks indicate statistical significance: *, *P* < 0.05; **, *P* < 0.01; ***, *P* < 0.001. ns, not significant.

The results in Figure 1A showed significant reductions in the phenotype of the Heinong531 (ST) cultivar when grown in saline-alkali soil. Specifically, there were reductions in stem length (31%) (Figure 1A), effective pod number per plant (2.5%), number of grains per plant (24.5%), grain weight per plant (36.2%), 100 seed weight (6.6%), and yield per unit area (36.4%). Similarly, the 20_1846 (SS) cultivar exhibited a decrease in phenotype under saline-alkali conditions, with reductions observed in stem length (54.4%), bottom pod height (3.6%), effective pod number per plant (21.5%), number of grains per plant (34.7%), grain weight per plant (34.1%), 100 seed weight (22.4%), and yield per unit area (22.2%). These findings indicate the negative impact of saline-alkali soil on the growth and productivity of both soybean cultivars. Eight indicators were measured, and the results showed that the saline-alkali soil had higher concentrations of HCO_3_^–^ (83.2), Cl^–^ (47.8%), Mg^2+^ (51%), SO_4_^2–^ (36.7), and Na^+^ (51.3) than normal soil, which was richer in Ca^2+^ (31.1%) and K^+^ (74.1%) (Figure 1B). Redundancy analysis (RDA) revealed that much of the phenotype in saline-alkali soil was associated with pH and ion (HCO_3_^–^, Cl^–^, Mg^2+^, SO_4_^2–^, and Na^+^), while the phenotype in normal soil was correlated with Ca^2+^ and K^+^ (Figure 1C). To better understand the correlation between phenotype and ions, the Pearson correlation coefficient was used to determine the relationship between the phenotype of soybean (SL, EPNPP, NGPP, GWPP, GW100, and PMY) and various ions (Ca^2+^, K^+^, HCO_3_^–^, Cl^–^, Mg^2+^, SO_4_^2–^, Na^+^, and pH). As shown in Figure 1D the results showed that there was a significant positive correlation between the phenotype of soybean and ions Ca^2+^ and K^+^, while a significant negative correlation was observed between the phenotype of soybean and ions HCO_3_^–^, Cl^–^, Mg^2+^, SO_4_^2–^, Na^+^, and pH. The results of the RDA analysis showed that the phenotype and yield of soybeans were strongly affected by the concentration of ions in the soil. It was found that an abundance of ions such as HCO_3_^–^, Cl^–^, Mg^2+^, SO_4_^2–^, and Na^+^ in combination with a high pH level caused a decrease in plant height and yield when grown in saline-alkali soil.

### 3.2. Dynamic changes of the soil microorganism between saline-alkali and normal soil

**FIGURE 2:**
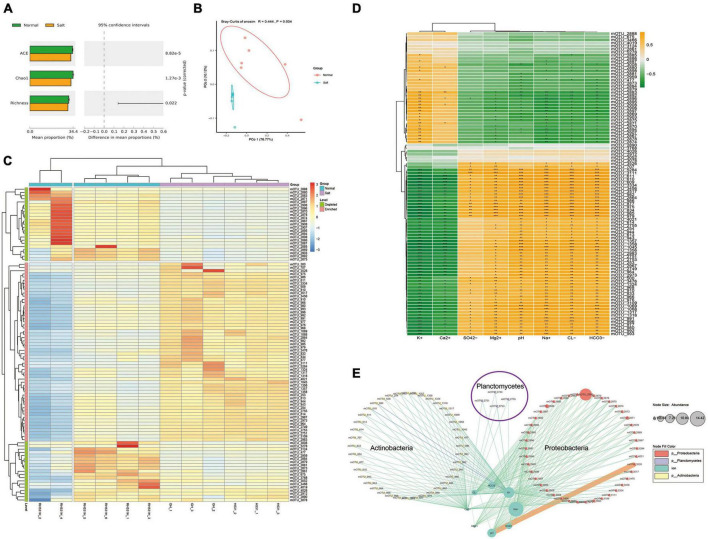
Dynamic changes of the soil microorganism between salinity and normal soil. **(A)** Effects of salinity and normal on rhizosphere soil microorganism ACE, Chao1, and Richness index (*p* < 0.05). **(B)** Principal-coordinate analysis (PCoA) and analysis of similarities (ANOSIM) based on Bray-Curtis’s dissimilarities showing differences in rhizosphere microorganism community structure under the normal and salinity soil (*R* = 0.444, *P* = 0.004). **(C)** Clustered heat map shows the differential species based on edgeR analysis, filter conditions (*p*-value < 0.05, FDR < 0.2), and 98 differential mOTUs were obtained. **(D)** Correlation analysis between differential mOTUs and ion concentration showed in clustered heat map. Yellow boxes represent positive correlations, while green boxes represent negative correlations (Pearson’s correlation). Black asterisks indicate statistical significance: *, *P* < 0.05; **, *P* < 0.01; ***, *P* < 0.001. **(E)** A co-occurrence network constructed and display the correlation of differential mOTUs and ion concentration.

The research utilized a metagenomic approach, where each sample was subjected to sequencing, resulting in 10 Gb of raw data per sample. Overall, a total of 120 GB of raw data was obtained from the study. Through analysis using the Kraken 2 method, a comprehensive list of species was generated. The analysis identified a total of 5,673 species, comprising 5,264 bacteria, 236 archaea, 57 fungi, and 116 viruses. Then the species were further categorized into mOTUs (microbial operational taxonomic units) at the species level. This categorization provided a convenient way to manage and analyze the data for statistical purposes. To explore rhizosphere microorganisms of soybean microbial community differences in saline-alkali soil, the α-diversity (ACE, Chao1, and Richness) of the microbial community in each group was estimated (Figure 2A). The results showed that ACE, Chao1, and Richness index values in normal soil were significantly higher than those in the saline-alkali soil (*P*-value < 0.05). For the β-diversity analysis, the principal-coordinate analysis (PCoA) and analysis of similarities (ANOSIM) based on the Bray-Curtis distance method showed that the soil microorganism community structures under stress of saline-alkali were significantly distinguishable from those of the normal conditions (*R* = 0.444, *P* = 0.004, Figure 2B). Significant differences were observed between the microbial structures of the Salt and Normal soil, respectively (edger method, FDR < 0.05, Figure 2C). There were 42 mOTUs depleted and 56 mOTUs enriched in salt group relative to normal group. Moreover, we focus on ion-associated microbes by the Pearson correlation method (Figure 2D). We obtained 85 mOTUs in total with significant correlation with ions, inclined 22 mOTUs with significant positive correlation and 54 mOTUs with significant negative correlation with K^+^, 5 mOTUs with significant positive correlation and 35 mOTUs with significant negative correlation with Ca^2+^, 34 mOTUs with significant positive correlation and 1 mOTUs with significant negative correlation with SO42-, 51 mOTUs with significant positive correlation and 15 mOTUs with significant negative correlation with Mg^2+^, 54 mOTUs with significant positive correlation and 19 mOTUs with significant negative correlation with PH, 56 mOTUs with significant positive correlation and 21 mOTUs with significant negative correlation with Na^+^, 56 mOTUs with significant positive correlation and 22 mOTUs with significant negative correlation with Cl^–^, 56 mOTUs with significant positive correlation and 23 mOTUs with significant negative correlation with HCO3-. To investigate the potential reciprocal interactions between altered mOTU and ions, a co-occurrence network was constructed based on Pearson correlation analysis (Figure 2E). We found that 51 mOTUs that belong to Proteobacteria formed a strong co-occurring relationship with ions (HCO_3_^–^, Cl^–^, Mg^2+^, SO_4_^2–^, Na^+^) and pH, 41 mOTUs that belong to Actinobacteria formed a strong co-occurring relationship with ions (K^+^ and Ca^2+^), 4 mOTUs (mOTU_2755, mOTU_2753, mOTU_275, and OTU_2749) that belong to Planctomycetes formed a strong co-occurring relationship with ions (K^+^ and Ca^2+^).

### 3.3. The difference in rhizosphere species between salt-tolerant and sensitive varieties under saline-alkali soil

**FIGURE 3:**
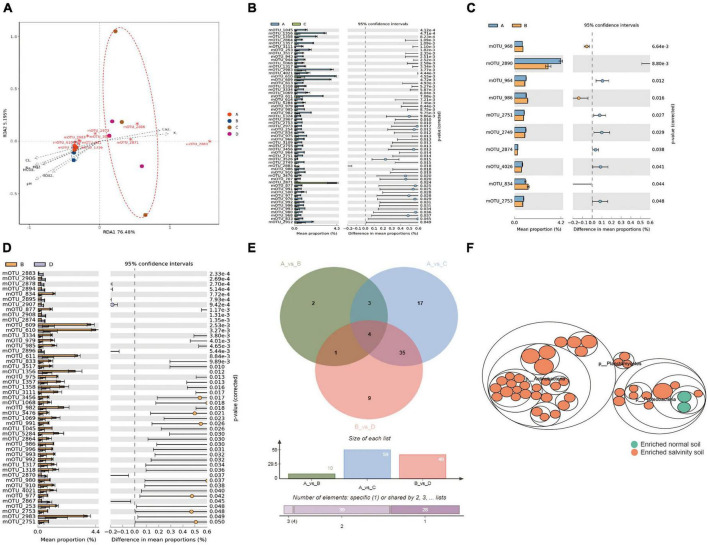
The difference of rhizosphere species between salt-tolerant and sensitive varieties under salinity stress. **(A)** Redundancy analysis (RDA) based on the microorganism community compositions of samples under the salinity soil (Mantel test, *n* = 12, *P* < 0.05). **(B)** The STAMP analysis the rhizosphere microbial difference of salt-tolerant varieties planted in salinity soil and normal soil. **(C)** STAMP analysis the rhizosphere microbial difference between salt-tolerant varieties planted in the salinity soil. **(D)** The rhizosphere microbial differences of sensitive varieties planted in salinity soil and normal soil. **(E)** Venn analysis of the mOTUs that significantly differed in relative abundance between comparisons of AvsC, AvsB, and BvsD. Clustered heat map shows a total of 43 mOTUs with significant differences were obtained which could be used as a biomarker of rhizosphere microorganisms in salinity soil and normal soil. Functional differences are based on the metagenome sequence data and assigned to taxonomic groups. **(F)** Taxonomic differences are based on metagenome data. The largest circles represent phylum level, and the inner circles represent class, family, genus, and species. Yellow circles represent enriched, while green circles represent depleted in salinity soil based on edgeR method with FDR < 0.05. Asterisks indicate statistical significance: *, *P* < 0.05; **, *P* < 0.01; ***, *P* < 0.001.

To further explore the key microorganisms of soybean rhizosphere in saline-alkali soil and normal soil. The samples were divided into four groups, the Salt-tolerant cultivar on the saline-alkali soil (A group), the Salt-sensitive cultivar on the saline-alkali soil (B group), the Salt-tolerant cultivar on the normal soil (C group) and the Salt-sensitive cultivar on the normal soil (D group). Redundancy analysis (RDA) clearly revealed that most of mOTUs in saline-alkali soil were associated with pH and ion (HCO_3_^–^, Cl^–^, Mg^2+^, SO_4_^2–^, and Na^+^), while the mOTUs in normal soil were correlated with Ca^2+^ and K^+^ (Figure 3A). When comparing the salt-tolerant cultivar in group (A) with the group (C), a total of 59 mOTUs were identified to have significant differences between the saline-alkali soil and normal soil (Figure 3B); A group compare with B group, both in the saline-alkali soil, recorded 10 mOTUs with significant differences (Figure 3C); Salt-sensitive variety in B group compare with D group, exhibited 49 mOTUs with significant differences between the saline-alkali soil and normal soil (Figure 3D). Through Venn diagram analysis (VDA) of three comparative studies, it was determined that a set of 43 key mOTUs (microbial operational taxonomic units) showed significant differences between groups A and B in the saline-alkali soil (Figure 3E). Metagenome data showed that, Planctomycetes, Proteobacteria and Actinobacteria dominated the difference bacterial community in the saline-alkali soil, with 29 mOTUs belong to Actinobacteria, 11 mOTUs belong to Proteobacteria and 3 mOTUs belong to Planctomycetes, all of which became enriched in the saline-alkali soil compared with the normal soil (Figure 3F). So far, we have obtained 43 key mOTUs which significantly correlation with ions (HCO_3_^–^, Cl^–^, Mg^2+^, SO_4_^2–^, Na^+^, K^+^, and Ca^2+^) and PH as biomarkers for the saline-alkali soil.

### 3.4. Dynamic changes of the function with the key microorganism in the C, N, P, and S cycle database

**FIGURE 4:**
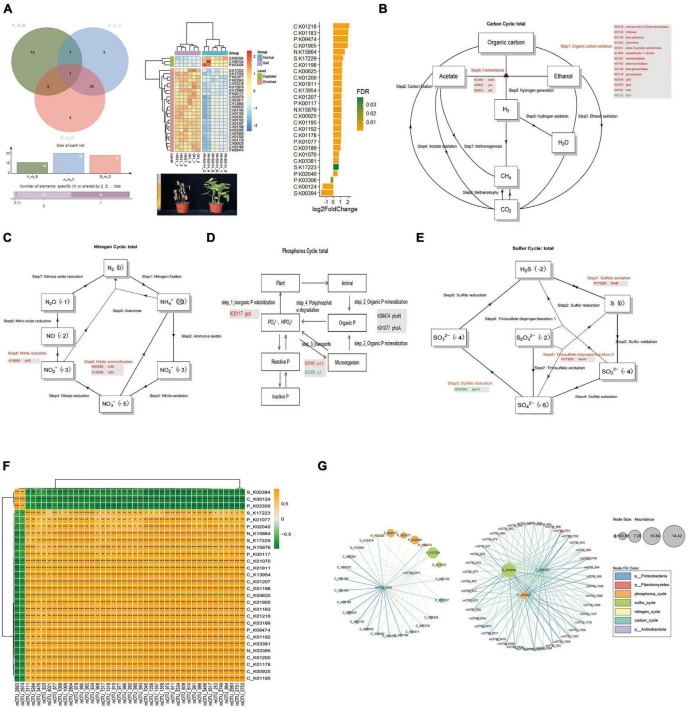
The CNPS function annotation and difference analysis. **(A)** Venn analysis of the KOs that significantly differed in relative abundance between comparisons of AvsC, AvsB, and BvsD. The heat map shows a total of 28 KOs with significant differences obtained which could use as the CNPS pathway biomarker of rhizosphere microorganisms in salinity soil and normal soil. **(B)** Carbon Cycle and critical key genes. **(C)** Nitrogen Cycle and critical key genes. **(D)** Phosphorus Cycle and critical key genes. **(E)** Sulfur Cycle and critical key genes. **(F)** Correlation analysis between differential mOTUs and key KOs. **(G)** Enrichment analysis of the differential mOTUs and key KOs. Yellow boxes represent positive correlations, while green boxes represent negative correlations (Pearson’s correlation). Black asterisks indicate statistical significance: *, *P* < 0.05; **, *P* < 0.01; ***, *P* < 0.001.

We observed significant shifts in specific microbial taxa in response to salt and alkali conditions. Among the notable taxa, Actinobacteria, Proteobacteria, and Planctomycetes exhibited the most pronounced shifts in abundance in the saline-alkali soil compared to normal soil. These shifts suggest a potential role of these microbial groups in responding to and potentially mitigating the challenges posed by high salt and alkaline levels. The enrichment of these taxa highlights their adaptive strategies and functional contributions to the soybean rhizosphere under stress conditions. To gain insight into the functioning of the soybean rhizosphere key microbiome, the metagenome data was compared to four databases [C cycling database (CCycDB), N cycling database NCycDB, P cycling database (PCycDB) and S cycling database (SCycDB)] to obtain functional gene annotation information. A total of 141 KEGG orthologs (Kos) were identified, including 66 KOs related to the carbon cycle, 16 KOs related to the nitrogen cycle, 48 KOs associated with the phosphorus cycle, and 11 KOs linked to the sulfur cycle. Using Venn diagram analysis (VDA), a total of 28 key significantly different KOs were obtained from three comparative studies. Of these, 25 KOs were enriched, and 3 KOs were depleted in response to the function of microorganisms in saline-alkali soil. These 28 KOs were found to be distributed across four different cycles: carbon, nitrogen, phosphorus, and sulfur. In the carbon cycle, 14 KOs were in Step 1 (Organic Carbon Oxidation), of which 13 were enriched and one was depleted. Additionally, 3 KOs were enriched in Step 6 (Fermentation). In the nitrogen cycle, one KO was enriched in Step 5 (Nitrite Reduction), and two KOs were enriched in Step 8 (Nitrite Ammonification). In the phosphorus cycle, 5 KOs were enriched while no depletion occurred. Finally, 3 KOs were enriched in the sulfur cycle with no depletions observed (Figures 4A–C). In the phosphorus cycle, one KO was enriched in Step 1 (Inorganic P solubilization), two KOs enriched in Step 2 (Organic P mineralization), and two KOs enriched and depleted in Step 3 (Transports) (Figure 4D). In the sulfur cycle, one KO was enriched in Step 1 (Sulfide oxidation), one KO was depleted in Step 5 (Sulfate reduction), and one KO was enriched in Step 9 (Thiosulfate disproportionation 2) (Figure 4E). Furthermore, by using the Pearson correlation method to focus on key KOs of biomarker microbes, two mOTUs (mOTU_2883 and mOTU_2874) were significantly positive correlation with 3 KOs (S_K00394, C_K00124, and P_K03306) and negative correlation with 25 KOs which 16 KOs (C_K00625, C_K00925, C_K01070, C_K01178, C_K01183, C_K01192, C_K01195, C_K01198, C_K01200, C_K01207, C_K01218, C_K01811, C_K01905, C_K03186, C_K03381, and C_K13954) in carbon cycle, 3 KOs (N_K03385, N_K15864, and N_K15876) in nitrogen cycle, 4 KOs (P_K00117, P_K01077, P_K02040, and P_K09474) in phosphorus cycle and 2 KOs (S_K17223 and S_K17229) in sulfur cycle, significantly positive correlation with 41 mOTUs (belong to Actinobacteria, Proteobacteria and Planctomycetes) (Figures 4F,G). We define that MB1 (microbial 1) which include 2 mOTUs (mOTU_2883 and mOTU_2874), MB2 which include 41 mOTUs (mOTU_3111, mOTU_5284, mOTU_3476, mOTU_833, mOTU_4021, mOTU_877, mOTU_1068, mOTU_1069, mOTU_ 2864, mOTU_979, mOTU_985, mOTU_982, mOTU_834, mOTU_968, mOTU_1317, mOTU_1318, mOTU_910, mOTU_ 977, mOTU_996, mOTU_992, mOTU_980, mOTU_993, mOTU_ 1045, mOTU_1356, mOTU_1357, mOTU_1358, mOTU_975, mOTU_611, mOTU_3334, mOTU_609, mOTU_610, mOTU_991, mOTU_986, mOTU_3456, mOTU_3517, mOTU_253, mOTU_ 2749, mOTU_964, mOTU_2983, mOTU_2751, and mOTU_2753), GC1 (gene cluster 1) which include 3 KOs (S_K00394, C_K00124 and P_K03306), GC2 which include 25 KOs (S_K17223, P_K01077, P_K02040, N_K15864, S_K17229, N_K15876, P_K00117, C_K01070, C_K01811, C_K13954, C_K01207, C_K01198, C_K00625, C_K01905, C_K01183, C_K01218, C_K03186, P_K09474, C_K01192, C_K03381, N_K03385, C_K01200, C_K01178, C_K00925, and C_K01195), the MB1 was significantly positive correlation with GC1 and negative correlation with GC2, but MB2 was the exact opposite of MB1.

### 3.5. Association analysis between key microorganisms and soybean phenotype

**FIGURE 5:**
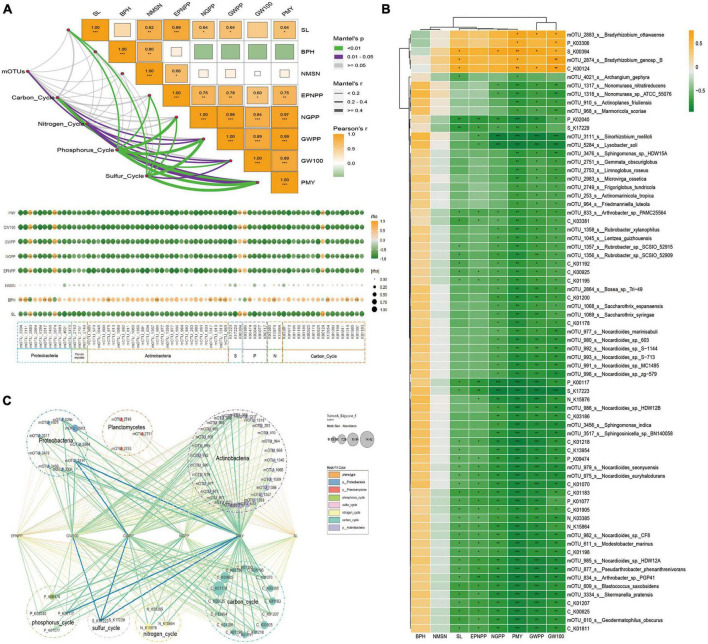
Effects of salt-relative microorganisms on soybean phenotype. **(A)** Correlations among soil ions and phenotype, function of the microbial data. The pairwise comparisons of ion concentration were shown, with a color gradient denoting Pearson’s correlation coefficient. Taxonomic community composition was related to each ion by the partial Mantel tests. The curve width is proportional to the Mantel’s r statistic for the corresponding distance correlations, and the curve color (see the legend on the right) denotes the statistical significance based on Pearson’s *p*-value **(B)** Correlation analysis among the phenotype, biomarker mOTUs, and KOs. Yellow boxes represent positive correlations, while green boxes represent negative correlations (Pearson’s correlation). Black asterisks indicate statistical significance: *, *P* < 0.05; **, *P* < 0.01; ***, *P* < 0.001. **(C)** Sankey diagram representing the contributions of phenotype to taxonomic mOTUs and functional genes. The six columns (from left to right) were values of Pearson’s *p*-value < 0.05.

The Mantel tests were used to assess the correlation among key microbial, function, and phenotype. The results showed that the mOTUs were significantly correlated with PMY, a function of the carbon cycle was correlated with the phenotype (PMY, GW100, and GWPP), a function of the nitrogen cycle was correlated with the phenotype (PMY, GW100, and GWPP), a function of phosphorus cycle was correlated with the phenotype (PMY, GW100, GWPP, NGPP, EPNPP, and SL), a function of the sulfur cycle was correlated with the phenotype (PMY, GW100, GWPP, NGPP, EPNPP, and SL) (Figure 5A). All the key microbial functions had not significantly correlation with BPH and NMSN. SL was significantly positive correlation with mOTU_2874 (belong to Proteobacteria) and negative correlation with 14 mOTUs (belong to Actinobacteria and Proteobacteria), EPNPP was significantly negative correlation with 13 mOTUs (belong to Actinobacteria and Proteobacteria), NGPP was significantly negative correlation with 31 mOTUs (belong to Actinobacteria and Proteobacteria), GWPP was significantly positive correlation with mOTU_2883 (belong to Proteobacteria) and negative correlation with 40 mOTUs (belong to Actinobacteria, Proteobacteria and Planctomycetes), GW100 was significantly positive correlation with 2 mOTUs (belong to Proteobacteria) and negative correlation with 40 mOTUs (belong to Actinobacteria, Proteobacteria and Planctomycetes), PMY was significantly positive correlation with 2 mOTUs (belong to Proteobacteria) and negative correlation with 40 mOTUs (belong to Actinobacteria, Proteobacteria and Planctomycetes) (Figure 5B). To investigate the potential reciprocal interactions among altered microbial, function and phenotype, a co-occurrence network was constructed based on Pearson correlation analysis (Figure 5C). We found that all the key microbial function had not significantly correlated with BPH and NMSN. SL was significantly positive correlation with mOTU_2874 (belong to MB1) and negative correlation with 14 mOTUs (belong to MB2), EPNPP was significantly negative correlation with 13 mOTUs (belong to MB2), NGPP was significantly negative correlation with 31 mOTUs (belong to MB2), NGPP was significantly negative correlation with 31 mOTUs (belong to MB2), GWPP was significantly positive correlation with mOTU_2883 (belong to MB1) and negative correlation with 40 mOTUs (belong to MB2), 100 GW was significantly positive correlation with 2 mOTUs (belong to MB1) and negative correlation with 40 mOTUs (MB2), PMY was significantly positive correlation with 2 mOTUs (MB1) and negative correlation with 40 mOTUs (belong to MB2). These findings indicate that altered soil microbiota and phenotype formed a synergistic and node-related co-occurrence network between the Salt and Normal groups.

### 3.6. The difference in rhizosphere species between salt-tolerant and sensitive varieties under saline-alkali soil

Redundancy analysis (RDA) clearly revealed that many of mOTUs in saline-alkali soil were associated with pH and ion (HCO_3_^–^, Cl^–^, Mg^2+^, SO_4_^2–^ and Na^+^), while the mOTUs in normal soil were correlated with Ca^2+^ and K^+^ (Figure 3A). The same variety of salt-tolerant, A group (Salt-tolerant on the saline-alkali soil) compare with C group (Salt-tolerant on the normal soil), recorded 59 mOTUs with significant differences between the saline-alkali soil and normal soil (Figure 3B); A group compare with B group (Salt-sensitive on the saline-alkali soil), both in the saline-alkali soil, showed 10 mOTUs with significant differences (Figure 3C); B group compare with D group (Salt-sensitive on the normal soil), exhibited 49 mOTUs with significant differences between the saline-alkali soil and normal soil (Figure 3D). Venn diagram analysis (VDA) of three comparative studies, revealed a total of 43 key significantly difference mOTUs to distinguish A and B group in the saline-alkali soil (Figure 3E). Metagenome data showed that Planctomycetes, Proteobacteria and Actinobacteria dominated the difference bacterial community in the saline-alkali soil, with 29 mOTUs belong to Actinobacteria, 11 mOTUs belong to Proteobacteria and 3 mOTUs belong to Planctomycetes, all of which became enriched in the saline-alkali soil compared with the normal soil (Figure 3F). So far, 43 key mOTUs significantly correlation with ions (HCO_3_^–^, Cl^–^, Mg^2+^, SO_4_^2–^, Na^+^, K^+^, and Ca^2+^) and PH as biomarkers for the saline-alkali soil.

### 3.7. Dynamic changes of the function with the key microorganism in the CNPS cycle

To gain insight into the functioning of the soybean rhizosphere key microbiome, the metagenome data was compared to four databases (CCycDB, NCycDB, PCycDB, and SCycDB) to obtain functional gene annotation information. A total of 141 KOs were identified, including 66 KOs related to the carbon cycle, 16 KOs related to the nitrogen cycle, 48 KOs associated with the phosphorus cycle, and 11 KOs linked to the sulfur cycle. Using Venn diagram analysis (VDA), a total of 28 key significantly different KOs were obtained from three comparative studies. Of these, 25 KOs were enriched, and 3 KOs were depleted in response to the function of microorganisms in saline-alkali soil. These 28 KOs were found to be distributed across four different cycles: carbon, nitrogen, phosphorus, and sulfur. In the carbon cycle, 14 KOs were in Step 1 (Organic Carbon Oxidation), of which 13 were enriched and one was depleted. Additionally, 3 KOs were enriched in Step 6 (Fermentation). In the nitrogen cycle, one KO was enriched in Step 5 (Nitrite Reduction), and two KOs were enriched in Step 8 (Nitrite Ammonification). In the phosphorus cycle, 5 KOs were enriched while no depletion occurred. Finally, 3 KOs were enriched in the sulfur cycle with no depletions observed (Figures 4A–C and Supplementary Table 1). In the phosphorus cycle, one KO was enriched in Step1 (Inorganic P solubilization), two KOs enriched in Step 2 (Organic P mineralization), and two KOs enriched and depleted in Step 3 (Transports) (Figure 4D). In the sulfur cycle, one KO was enriched in Step 1 (Sulfide oxidation), one KO was depleted in Step 5 (Sulfate reduction), and one KO was enriched in Step 9 (Thiosulfate disproportionation 2) (Figure 4E). Furthermore, by using the Pearson correlation method to focus on key KOs of biomarker microbes, two mOTUs (mOTU_2883 and mOTU_2874) were significantly positive correlation with 3 KOs (S_K00394, C_K00124 and P_K03306) and negative correlation with 25 KOs which 16 Kos (C_K00625, C_K00925, C_K01070, C_K01178, C_K01183, C_K01192, C_K01195, C_K01198, C_K01200, C_K01207, C_K01218, C_K01811, C_K01905, C_K03186, C_K03381, and C_K13954) in carbon cycle, 3 Kos (N_K03385, N_K15864, and N_K15876) in nitrogen cycle, 4 KOs (P_K00117, P_K01077, P_K02040, and P_K09474) in phosphorus cycle and 2 Kos (S_K17223 and S_K17229) in sulfur cycle, significantly positive correlation with 41 mOTUs (belong to Actinobacteria, Proteobacteria and Planctomycetes) (Figures 4F,G). We define that MB1 (microbial 1) which include 2 mOTUs (mOTU_2883 and mOTU_2874), MB2 which include 41 mOTUs (mOTU_3111, mOTU_5284, mOTU_3476, mOTU_833, mOTU_4021, mOTU_877, mOTU_1068, mOTU_1069, mOTU_ 2864, mOTU_979, mOTU_985, mOTU_982, mOTU_834, mOTU_968, mOTU_1317, mOTU_1318, mOTU_910, mOTU_ 977, mOTU_996, mOTU_992, mOTU_980, mOTU_993, mOTU_ 1045, mOTU_1356, mOTU_1357, mOTU_1358, mOTU_975, mOTU_611, mOTU_3334, mOTU_609, mOTU_610, mOTU_991, mOTU_986, mOTU_3456, mOTU_3517, mOTU_253, mOTU_ 2749, mOTU_964, mOTU_2983, mOTU_2751, and mOTU_2753), GC1 (gene cluster 1) which include 3 KOs (S_K00394, C_K00124 and P_K03306), GC2 which include 25 KOs (S_K17223, P_K01077, P_K02040, N_K15864, S_K17229, N_K15876, P_K00117, C_K01070, C_K01811, C_K13954, C_K01207, C_K01198, C_K00625, C_K01905, C_K01183, C_K01218, C_K03186, P_K09474, C_K01192, C_K03381, N_K03385, C_K01200, C_K01178, C_K00925, and C_K01195), the MB1 was significantly positive correlation with GC1 and negative correlation with GC2, but MB2 was the exact opposite of MB1.

### 3.8. Association analysis between key microorganisms and soybean phenotype

The Mantel tests were used to assess the correlation among key microbes, function, and phenotype. The results showed that the mOTUs were significantly correlated with PMY, function of carbon cycle was correlated with phenotype (PMY, GW100, and GWPP), function of nitrogen cycle was correlated with phenotype (PMY, 100GW, and GWPP), function of phosphorus cycle was correlated with phenotype (PMY, 100GW, GWPP, NGPP, EPNPP, and SL), function of sulfur cycle was correlated with phenotype (PMY, 100 GW, GWPP, NGPP, EPNPP, and SL) (Figure 5A and Supplementary Table 1). All of the key microbial and function had no significantly correlation with BPH and NMSN, SL was significantly positive correlation with mOTU_2874 (belong to Proteobacteria) and negative correlation with 14 mOTUs (belong to Actinobacteria and Proteobacteria), EPNPP was significantly negative correlation with 13 mOTUs (belong to Actinobacteria and Proteobacteria), NGPP was significantly negative correlation with 31 mOTUs (belong to Actinobacteria and Proteobacteria), GWPP was significantly positive correlation with mOTU_2883 (belong to Proteobacteria) and negative correlation with 40 mOTUs (belong to Actinobacteria, Proteobacteria and Planctomycetes), 100 GW was significantly positive correlation with 2 mOTUs (belong to Proteobacteria) and negative correlation with 40 mOTUs (belong to Actinobacteria, Proteobacteria and Planctomycetes), PMY was significantly positive correlation with 2 mOTUs (belong to Proteobacteria) and negative correlation with 40 mOTUs (belong to Actinobacteria, Proteobacteria and Planctomycetes) (Figure 5B). To investigate the potential reciprocal interactions among altered microbial, function and phenotype, a co-occurrence network was constructed based on Pearson correlation analysis (Figure 5C). We found that all of the key microbial and function had no significantly correlation with BPH and NMSN, SL was significantly positive correlation with mOTU_2874 (belong to MB1) and negative correlation with 14 mOTUs (belong to MB2), EPNPP was significantly negative correlation with 13 mOTUs (belong to MB2), NGPP was significantly negative correlation with 31 mOTUs (belong to MB2), NGPP was significantly negative correlation with 31 mOTUs (belong to MB2), GWPP was significantly positive correlation with mOTU_2883 (belong to MB1) and negative correlation with 40 mOTUs (belong to MB2), 100GW was significantly positive correlation with 2 mOTUs (belong to MB1) and negative correlation with 40 mOTUs (MB2), PMY was significantly positive correlation with 2 mOTUs (MB1) and negative correlation with 40 mOTUs (belong to MB2). These findings indicate that altered soil microbiota and phenotype formed a synergistic and node-related co-occurrence network between the Salt and Normal groups.

## 4. Discussion

**FIGURE 6:**
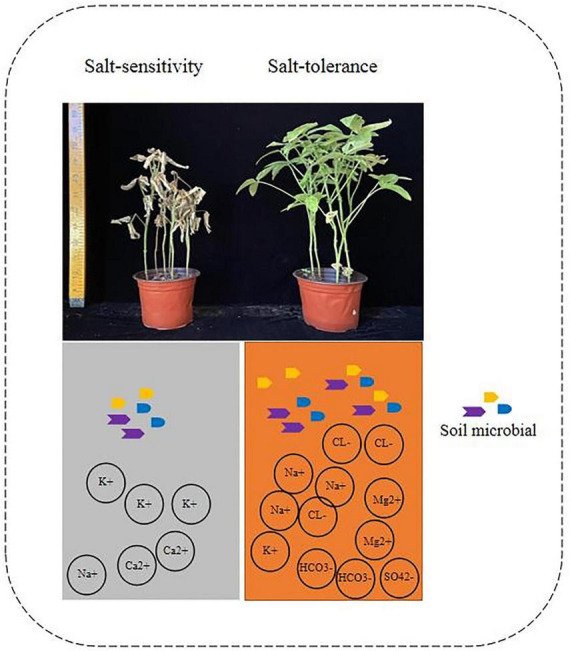
Relationship between rhizosphere microorganisms and salt ion concentration of salt-tolerant varieties.

In this study, two cultivars: salt-tolerant and salt-sensitive were used to examine the resistance of soybean plants to salt and alkali conditions. The enhancement of plant growth is intricately linked to intricate and diverse changes in the composition and dynamics of rhizosphere microbial communities (Kinkel et al., 2011; Mendes et al., 2011; Fu et al., 2017). These changes are strongly influenced by specific microbial populations that play a crucial role in supporting plant development, known as potential key rhizosphere microbial taxa (Kinkel et al., 2011; Bandopadhyay et al., 2020). By integrating and analyzing the sequencing data obtained from rhizosphere microbiota, we can gain valuable insights into the significant role played by these specialized microbial taxa in the overall life of plants (Berry, 2016; Bandopadhyay et al., 2020). Our study revealed the key role of the rhizosphere microbial effect on soybean phenotype, as demonstrated by four groups of soybean plants grown in Salt and Normal soil. We found that the soybean phenotype was significantly correlated with soil salt ion concentration, with salt-tolerant varieties having higher concentrations of Na^+^, Mg^+^, HCO_3_^–^, Cl^–^, and SO_4_^2–^ (Figure 6). The results of this study highlight the significant negative impact of saline-alkali soil on soybean growth. The reduction in various soybean phenotypic traits, such as stem length, pod height, pod number, grain weight, and seed weight, in saline-alkali soil compared to normal soil demonstrates the sensitivity of soybean plants to high concentrations of ions, particularly HCO_3_^–^, Cl^–^, Mg^2+^, SO_4_^2–^, Na^+^, and elevated pH levels. These findings are consistent with previous research indicating that excess salts and alkaline conditions in the soil can hinder plant growth and yield (Zörb et al., 2019). Previous research has indicated that the roots of salt-tolerant varieties tend to have higher levels of Na^+^ and lower levels of K^+^, while normal plants have higher levels of K^+^ and lower levels of Na^+^ (Lian et al., 2020; Sola-Leyva et al., 2021). The lack of potassium (K^+^) in plants can be attributed to intense competition between consumed sodium chloride (NaCl) and other minerals, particularly K^+^ (Ball and Farquhar, 1984). However, a decrease in K levels was observed only in the “Black Beauty” variety at higher salt concentrations. As a result, the ratios of Na/K and Na/Ca were lower in the “Bonica” variety compared to “Black Beauty” (Perez-Alfocea et al., 1996; Yasar et al., 2006). These ionic ratios play a significant role in determining the salt susceptibility and tolerance of plants. Cultivars that are tolerant to salt stress typically exhibit lower Na/K and Na/Ca ratios. Maintaining a low cytosolic Na/K ratio is crucial for plants to achieve optimal growth, particularly at the cellular level. When salinity increases, Na competes with K for uptake, potentially inhibiting K-specific transporters (Zhu, 2003; Yasar et al., 2006). This leads to a toxic accumulation of Na and a deficiency of K, which affects osmotic regulation and enzyme stability. To adapt to salt stress, plants have developed various mechanisms, including regulating the consumption of Na at the cellular level and transporting Na over long distances. Understanding the importance of these specialized rhizosphere microbial taxa in plant life can be achieved through the integration (Munns et al., 2000) and analysis of rhizosphere microbiota sequencing data Also the composition of the rhizosphere microbiome of legumes is dependent on the genotype of the host (Mendes et al., 2014; Miranda-Sánchez et al., 2016; Hartman et al., 2017; Zhong et al., 2019). Our findings demonstrate that soybean plants grown in different types of soil have a greater microbial diversity in their rhizosphere than in their roots (Figure 2), which is consistent with previous research conducted on soybean and alfalfa (Xiao et al., 2017).

The analysis of rhizosphere microorganisms revealed notable differences between saline-alkali and normal soil. The lower alpha-diversity indices (ACE, Chao1, and Richness) in the saline-alkali soil suggest a reduced microbial community abundance and diversity, indicating the adverse impact of salt-alkaline stress on microbial populations. The significant dissimilarities in microbial community structures between saline-alkali and normal soil further support the notion that soil conditions strongly influence microbial composition. The enrichment of certain microbial taxa, including Actinobacteria, Proteobacteria, and Planctomycetes, in the saline-alkali soil suggests their potential roles in adapting to and thriving under adverse soil conditions. Furthermore, the study revealed strong correlations between soil ion concentrations, key microbial taxa, functional gene composition, and soybean phenotypes. The positive correlation of soybean phenotypes with Ca^2+^ and K^+^ ions in normal soil underscores the importance of these ions for promoting plant growth and yield. Conversely, the negative correlation of soybean phenotypes with HCO_3_^–^, Cl^–^, Mg^2+^, SO_4_^2–^, Na^+^, and pH in saline-alkali soil highlights the detrimental effects of these ions and high pH levels on plant development. In normal soil, ions like Ca^2+^ and K^+^ positively correlate with soybean growth due to their essential roles in plant physiological processes. Calcium (Ca^2+^) is critical for cell division, membrane stability, and signal transduction, while potassium (K^+^) is involved in osmotic regulation and enzyme activation (Meriño-Gergichevich et al., 2010). On the contrary, in saline-alkali soil, ions like HCO_3_^–^, Cl^–^, Mg^2+,^ SO_4_^2–^, Na^+^, and elevated pH exhibit negative correlations with soybean growth. High levels of these ions and alkaline pH can disrupt nutrient uptake, interfere with ion balance, and impede enzyme activity, leading to reduced plant health and growth (Phang et al., 2008).

Co-occurrence interactions observed through the co-occurrence network analysis have significant implications for nutrient cycling, community stability, and pathogen suppression in plant-microbe interactions (Zhang et al., 2018). The intricate relationships between specific microbial taxa, ions, and functional genes suggest a collaborative network where key microorganisms contribute to nutrient-cycling processes, such as organic carbon oxidation and phosphorus mineralization, crucial for soil fertility and plant growth (Mendes et al., 2014). The observation that the microbial community in the roots is more resilient to environmental changes implies that these root-associated microbes play a vital role in maintaining the stability and resilience of the plant-soil system. The ability of legumes, like soybeans, to selectively attract specific microbes underscores the potential for promoting beneficial interactions, such as nitrogen fixation and growth promotion (Trivedi et al., 2020). Additionally, the strong correlations among microorganisms in the rhizosphere can influence pathogen suppression through competitive exclusion, resource competition, and the production of antimicrobial compounds. These co-occurrence interactions offer insights into mechanisms that can enhance nutrient availability, ecosystem stability, and disease resistance, crucial for sustainable agricultural practices (Miranda-Sánchez et al., 2016; Zhong et al., 2019). Our findings demonstrated strong correlations among microorganisms within the rhizome compartment, particularly in the rhizosphere (Figure 3). These results align with a study conducted on 51 soybean fields across China, which also concluded that bacterial subnetworks in both bulk soil and the rhizosphere were significantly influenced by soil pH (Zhang et al., 2018).

These microbial networks provide insights into the co-occurrence patterns and interactions among microorganisms, which have the potential to impact the composition of microbial communities and their interactions with host plants. Furthermore, our study identified 43 salt-tolerant species belonging to Actinobacteria, Proteobacteria, and Planctomycetes. Furthermore, other ecosystems with microbial communities that possess similar metabolic pathways, such as tropical peatlands or coastal salt marshes, may also contain TMO-producing Planctomycetes, thus allowing these lipids to be used to understand microbial community responses to environmental change in a variety of systems (Moore, 2021). The salt marsh microbial communities were found to be dominated by three phyla: Alpha proteobacteria (15%), Gamma proteobacteria (17%), and Planctomycetes (11%). These phyla were observed to have increased abundances after repeated salinity shock-recovery phases, indicating their ability to adapt to NaCl shock and recovery (He et al., 2019; Tebbe et al., 2022). Among the 43 key mOTUs identified, 29 belonged to Actinobacteria, 11 belonged to Proteobacteria and 3 belonged to Planctomycetes. Additionally, 28 key KOs were identified, of which 17 were involved in the carbon cycle, 3 in the nitrogen cycle, 5 in the phosphorus cycle, and 3 in the sulfur cycle. We found that the 16 KOs focus on and enriched in Organic carbon oxidation and Fermentation pathway in the C cycle, this indicates that in saline-alkali soil, salt-tolerant bacteria promote the oxidation of organic carbon, resulting in the reduction of soil organic carbon, resulting in soil fertility and unfavorable crop growth. Increased soil organic carbon can increase crop yields (Amoah et al., 2022). Increasing organic carbon storage stock for sustainable agriculture (Yang et al., 2023). There were 3 KOs enriched in Nitrite reduction and Nitrite ammonification pathway in the N cycle, this indicates that in saline-alkali soil, salt-tolerant bacteria promote soil nitrogen (N) loss (Meakin et al., 2007). Salt-tolerant bacteria in the cytosol and bacteroids of water-stressed soybean nodules were found to exhibit nitrate reduction. Additionally, five specific KOs (functional gene categories) associated with inorganic phosphorus solubilization, organic phosphorus mineralization, and transport were enriched, indicating that in saline-alkali soil, these bacteria may contribute to soil nitrogen loss. Moreover, the low availability of phosphorus in the soil was found to hinder plant growth, as mentioned in reference (McArthur and Knowles, 1992; Han et al., 2020). Furthermore, four KOs related to sulfide oxidation and thiosulfate disproportionation, along with two KOs depleted in the sulfate reduction pathway, were observed in the sulfur cycle. This suggests that salt-tolerant bacteria in saline-alkali soil may enhance sulfide oxidation while inhibiting sulfate reduction. These findings potentially explain the effects of salt-tolerant rhizosphere microorganisms on the phenotype and yield of soybeans per mu (a unit of area) (Norris et al., 2020). While the influence of microbial populations on soybean plants is evident, attributing phenotypic changes solely to microbial effects can be challenging. Other factors, including plant genetics, abiotic factors, and interactions with non-target microbes, also play roles in shaping plant performance. Additionally, the complexity of microbial communities and their functional redundancy can make it difficult to pinpoint specific mechanisms driving observed effects. Thus, careful experimental design and advanced techniques, such as gnotobiotic systems and metagenomics, are necessary to disentangle the direct contributions of microbial populations to plant health and productivity. Overall, the study’s findings offer practical applications for agricultural practices in soybean cultivation on saline-alkali soils. Insights into specific microbial interactions with soybean plants inform the selection of salt-tolerant cultivars, the development of microbial inoculants, and improved soil management strategies. This knowledge supports sustainable agriculture by enhancing nutrient cycling, disease resistance, and stress tolerance. Additionally, precision agriculture can benefit from site-specific adjustments based on microbial insights, promoting efficient resource utilization. these practical implications contribute to higher yields, reduced environmental impact, and the overall resilience of soybean crops in challenging soil conditions.

## 5. Conclusion

In conclusion, this research study examined the impact of saline-alkali soil on soybean growth and its associated rhizosphere microorganisms. The results demonstrated that soybean growth was significantly impaired in saline-alkali soil compared to normal soil conditions. The presence of higher concentrations of HCO^3–^, Cl^–^, Mg^2+^, SO_4_^2–^, and Na^+^ ions in saline-alkali soil, along with a high pH level, negatively affected soybean phenotype and yield. The study also revealed distinct differences in the microbial community composition between saline-alkali and normal soil, with reduced microbial diversity in saline-alkali conditions. Specific microbial taxa identified as biomarkers for saline-alkali soil, suggesting their potential role in adapting to and mediating plant responses to such conditions. Additionally, functional gene analysis highlighted the involvement of key genes in carbon, nitrogen, phosphorus, and sulfur cycles, which correlated with soybean phenotypes. Compared with normal soil, the rhizosphere microbial diversity of soybean in saline-alkali soil significantly reduced, and there were enriched Actinobacteria, Proteobacteria, and Planctomycetes, which were significantly correlated with soybean yield. The molecular ecological network analysis further revealed unseen co-occurrence patterns of the putative bacterial species (OTUs) associated with the soybean rhizosphere. Our research has revealed that the rhizosphere microbiota plays a significant role in the rhizobia-soybean symbiosis and in helping plants adapt to stressful conditions. This knowledge can also be used to develop new strategies for improving the efficiency of soybean production, such as increasing the availability of beneficial microorganisms in the rhizosphere. Finally, this knowledge can be used to improve soil health and fertility by promoting beneficial microorganisms in the rhizosphere. Further research is warranted to explore the potential applications of these findings in practical agricultural settings and to understand the long-term effects of microbial interventions on crop performance and soil sustainability.

## Data availability statement

The datasets presented in this study can be found in online repositories. The names of the repository/repositories and accession number(s) can be found below: https://www.ncbi.nlm.nih.gov/, PRJNA961035.

## Author contributions

HR: conceptualization and writing—review and editing. FZ and XZ: conceptualization and writing. SL: methodology. KZ: software. JW and BZ: review and editing. All authors contributed to the article and approved the submitted version.
